# Validity of central pain processing biomarkers for predicting the occurrence of oncological chronic pain: a study protocol

**DOI:** 10.1186/s12885-024-12455-8

**Published:** 2024-06-08

**Authors:** M. T. Carrillo-de-la-Peña, C. Fernandes, C. Castro, PAINLESS Consortium, Lara Rubal, Noelia Samartin-Veiga, David Yarnitzsky, Lars Arendt-Nielsen, Carsten Dahl, R. Medeiros

**Affiliations:** 1 Brain and Pain (BaP) Lab, Departamento de Psicoloxía Clínica y Psicobioloxía, Facultade de Psicoloxia, Universidade de Santiago de Compostela, Campus Vida, Santiago de Compostela, A Coruña 15782 Spain; 2 Faculty of Human and Social Sciences, University Fernando Pessoa, Praça 9 de Abril, 349, Porto, 4249-004 Portugal; 3 Faculty of Psychology and Education Sciences, Laboratory of Neuropsychophysiology, University of Porto, Rua Alfredo Allen, Porto, 4200-135 Portugal; 4 Molecular Oncology and Viral Pathology Group, Research Center of IPO (CI-IPOP) & RISE@CI-IPOP (Health Research Network), Portuguese Oncology Institute of Porto (IPO Porto)/Porto Comprehensive Cancer Center (Porto. CCC), R. Dr. António Bernardino de Almeida 865, Porto, 4200-072 Portugal; 5 School of Health, Polytechnic Institute of Porto, Rua Dr. António Bernardino de Almeida, 400, Porto, 4200-072 Portugal; 6 Unit of Anatomy, Department of Biomedicine, Faculty of Medicine, University of Porto, Alameda Prof. Hernâni Monteiro, Porto, 4200-319 Portugal; 7 Faculty of Medicine, University of Porto, Alameda Prof. Hernâni Monteiro, Porto, 4200-319 Portugal; 8 Abel Salazar Institute of Biomedical Sciences (ICBAS), University of Porto, R. Jorge de Viterbo Ferreira 228, Porto, 4050-313 Portugal; 9 Virology Service, Portuguese Oncology Institute of Porto (IPO Porto), Porto, Rua Dr. António Bernardino de Almeida, 865, 4200-072 Portugal; 10 Biomedical Research Center (CEBIMED), Faculty of Health Sciences of Fernando Pessoa University, Praça 9 de Abril, 349, Porto, 4249-004 Portugal

**Keywords:** Cancer pain, Chronic pain, EEG, QST, CHEPs

## Abstract

**Background:**

Despite recent improvements in cancer detection and survival rates, managing cancer-related pain remains a significant challenge. Compared to neuropathic and inflammatory pain conditions, cancer pain mechanisms are poorly understood, despite pain being one of the most feared symptoms by cancer patients and significantly impairing their quality of life, daily activities, and social interactions. The objective of this work was to select a panel of biomarkers of central pain processing and modulation and assess their ability to predict chronic pain in patients with cancer using predictive artificial intelligence (AI) algorithms.

**Methods:**

We will perform a prospective longitudinal cohort, multicentric study involving 450 patients with a recent cancer diagnosis. These patients will undergo an in-person assessment at three different time points: pretreatment, 6 months, and 12 months after the first visit. All patients will be assessed through demographic and clinical questionnaires and self-report measures, quantitative sensory testing (QST), and electroencephalography (EEG) evaluations. We will select the variables that best predict the future occurrence of pain using a comprehensive approach that includes clinical, psychosocial, and neurophysiological variables.

**Discussion:**

This study aimed to provide evidence regarding the links between poor pain modulation mechanisms at precancer treatment in patients who will later develop chronic pain and to clarify the role of treatment modality (modulated by age, sex and type of cancer) on pain. As a final output, we expect to develop a predictive tool based on AI that can contribute to the anticipation of the future occurrence of pain and help in therapeutic decision making.

## Background

The most recent data indicate that in 2020, an estimated 18.1 million new cancer cases occurred globally [43]. Despite recent improvements in cancer detection and survival rates, managing cancer-related pain remains a significant challenge. The first systematic review on the prevalence and severity of cancer pain revealed that 59% of patients undergoing cancer treatment, 64% of patients with advanced cancer, and 33% of patients who have been cured still suffer from pain [40]. Updated data from 2022 showed a decrease in both the prevalence and severity of pain over the past decade. However, even with this decline, the combined prevalence rates still resulted in an overall prevalence of 44.5%. The prevalence of pain is particularly high among patients with advanced, metastatic, and terminal cancer, reaching 54.6% of patients [31].

Chronic pain is a complex sensory and emotional experience that differs among individuals based on factors such as circumstances, personal perceptions, and physiological conditions. It is characterized by its persistence or recurrence, surpassing the anticipated period for natural healing (i.e., lasting more than three months; [37]) and, thereby, lacking the protective function of acute nociceptive pain [1, 16]. Although the causes and clinical symptoms of chronic pain vary, they may share common dysfunctional pain regulatory mechanisms.

Cancer-related pain is influenced by multiple factors, including peripheral inflammation, nerve damage, and spinal-level central sensitization. However, brain mechanisms also play a role. Evidence suggests that both inhibitory (anti-nociceptive) and facilitatory (pro-nociceptive) pathways are crucial for understanding chronic and neuropathic pain in cancer patients. Following the processing and modulation of nociceptive signals at the spinal level (dorsal horn neurons), various brain regions contribute to the sensory-discriminative (e.g., somatosensory cortex) and emotional aspects of pain (e.g., limbic structures such as the amygdala, insula, and cingulate cortex).

Conventional management of pain is based on a combination of pharmacological and nonpharmacological therapies, including oral nonsteroidal anti-inflammatory drugs, physical therapy, and opioid analgesics [13, 15]. These options may have some benefits, but they are often associated with significant adverse effects and/or limited treatment benefits over time (due to therapy tolerance, disease progression, and/or neural sensitization of pain-related neural structures; [3, 28]). When opioids fail, surgical interventions are not successful, or when drugs lead to adverse events on a consistent basis, it may be useful to consider alternative strategies. Compared to neuropathic and inflammatory pain states, cancer pain mechanisms are poorly understood [9], despite pain being one of the most feared symptoms by cancer patients, significantly impairing their quality of life, daily activities, and social interactions [36].

The limited effectiveness of pharmacological methods highlights the need to investigate alternative approaches to pain management in cancer patients. Comprehending the pain mechanisms in cancer patients is crucial for enhancing its management. Moreover, the failure of conventional treatments suggests the need to shift from a disease-oriented to a mechanism-based management strategy.

The current study sought to enhance the management of cancer-related pain by deepening our understanding of its fundamental mechanisms. Using a longitudinal approach, we explored diagnostic biomarkers obtained from quantitative sensory testing (QST) and brain electrical activity, which may elucidate the origins and persistence of pain and allow for patient stratification based on these mechanisms and clinical variables. Our approach aims to better understand the central mechanisms of cancer pain as a requisite for improving pain management.

The QST is a noninvasive, sensitive tool for describing the function of both antinociceptive and pronociceptive pathways. Specifically, through two validated paradigms—conditioned pain modulation (CPM) and temporal summation of pain (TSP)—it is possible to assess the dynamic mechanisms of central pain modulation. CPM is measured as the reduction in pain provoked by a noxious stimulus (test stimulus) when another painful stimulus (conditioning stimulus) is applied to a remote area (pain inhibits pain; [38]). TSP occurs when repeated noxious stimuli over the same corporal area amplify pain sensation [2].

Multiple studies have confirmed that many chronic pain patients tend to exhibit increased excitability in response to nociceptive stimuli and limited central analgesic regulation compared to healthy controls [10, 17, 26, 33, 34]. Several studies have shown greater TSP in chronic pain patients than in healthy controls [20, 24]. Approximately 70% of chronic pain patients with CPM display a large and statistically significant reduction in their pain inhibition system relative to that of healthy controls [20].

Although quantifying the function of descending pain modulatory pathways would improve our understanding of cancer pain, a recent systematic review highlighted the lack of QST data in cancer patients [22]. Nevertheless, there is some evidence suggesting the role of abnormal descending controls in cancer pain. For instance, compared with women who did not experience pain after surgery, women who experienced chronic pain after breast cancer surgery displayed enhanced TSP, mechanical pain and deficits in CPM [8]. These results suggested that persistent postoperative pain may be associated with alterations in endogenous pain inhibition in the central nervous system and that treatment strategies should target those pain-modulatory systems. Moreover, most of the studies reviewed included patients with chemotherapy-induced peripheral neuropathy and exhibited abnormal responses (mostly thresholds) to a variety of stimuli. Nevertheless, few studies have considered dynamic indices, such as CPM or TSP, which are more closely linked to the underlying neurobiological mechanism of pain. Additionally, this review underscores the small number of QST studies profiling cancer pain types.

While the QST allows the assessment of psychophysiological mechanisms of central pain modulation, the study of electroencephalography (EEG), in the resting state and in response to noxious stimuli, may also help to characterize brain responsiveness to nociceptive stimulation. The neural signatures of pain at the scalp level include modifications in oscillatory electrical activity (in frequencies such as theta, alpha, and gamma), which are related to activity in areas such as the operculo-insular cortex or the primary somatosensory cortex [25]. When a noxious stimulus is perceived as painful, power increases and decreases at those bands and locations. In addition, these frequencies (specifically theta and gamma) appear to be intimately related since the high-frequency gamma oscillations appear embedded in specific phases of theta, a mechanism related to the integrated perception of pain [21].

This knowledge about oscillatory activity during pain was almost exclusively drawn from the study of healthy populations, with practically no investigations of brain activity in cancer pain. Van den Brooke et al. [41] reported that patients who experienced persistent pain after breast cancer treatment exhibited increased alpha activity in spontaneous EEG signals compared with patients who also experienced breast cancer treatment but did not experience pain [41]. Interestingly, there have been attempts to characterize pain phenotypes by machine learning using EEG features [19], but thus far, this technique has not been applied to cancer pain.

In addition, indices of pain processing with excellent temporal resolution can be derived from EEGs. This is the case for contact heat-evoked potentials (CHEPs), which are obtained during the presentation of painful hot stimuli. CHEPs are negative waves observed over central-parietal scalp areas contralateral to the stimulated hand (N1 wave), and a biphasic negative–positive complex is maximal at the vertex and peaks from 190 to 400 ms (N2 and P2 waves) after stimulus presentation [5]. We have found no studies analyzing electrical brain activity during the presentation of nociceptive stimulation in patients with cancer. As a response to the gaps found, in this study, we will apply sophisticated analysis techniques (time–frequency and connectivity) to improve our knowledge about the mechanisms of pain processing or modulation and to stratify cancer pain patients using the proposed biomarkers.

In summary, previous research on central pain biomarkers has supported the use of QST indices as predictors of the occurrence of chronic pain. Preexisting sensory deficits prior to chemotherapy are a contributing factor to the onset of chemotherapy-induced peripheral neuropathy [40], and QST indices predict treatment response in patients with cancer-induced bone pain [31]. However, these data are scarce for validating the predictive power of those biomarkers. In addition, no specific bedside protocol for assessing central pain biomarkers or specific tools for cancer pain prediction have been developed thus far.

The main objective of this study was to select a panel of biomarkers of central pain processing and modulation (QST and EEG indices) to assess their validity in predicting chronic pain in patients with cancer considering demographic and clinical moderators (such as type of tumor, treatment modality, sex, age, clinical status, etc.). Moreover, we propose a bed-site assessment protocol and predictive artificial intelligence (AI) algorithms to predict the future occurrence of pain. We hypothesize that (H1) patients with poorer pain modulation mechanisms (larger TSP, lower CPM and greater evoked potential responses to noxious stimuli) at precancer treatment will have a greater probability of developing chronic pain 6 and 12 months after treatment onset; (H2) clinical (i.e., type of cancer and treatment) and personal variables (age, sex, body mass index) will be significant predictors of the occurrence of oncological pain; and (H3) it will be possible to develop a prognostic tool for oncological pain using AI algorithms.

## Methods

### Aims of the study

The primary objective of this clinical study was to select a panel of biomarkers of central pain processing and modulation and assess their ability to predict chronic pain in patients with cancer by proposing a bed-site assessment protocol and predictive AI algorithms (to anticipate the future occurrence of pain). To achieve this goal, we propose the following specific objectives:

- To select a set of tools to assess core domains in clinical trials on pain in palliative care patients (pain and comorbid symptoms, sleep quality, emotional state, cognitive functioning, medication intake, functionality, quality of life).
- To select a panel of biomarkers of central pain processing and modulation for use in a bed-site protocol assessment.
- The aim of this multicenter study was to standardize a protocol for assessment in clinical settings.
- To evaluate the predictive validity of all the biomarkers using AI analysis.

### Study design and population sample size

This was a prospective, naturalistic, longitudinal, cohort, multicentric study involving 450 patients screened according to the inclusion and exclusion criteria presented in Table 1. All these patients will be assessed at three time points (pretreatment, 6 months, and 12 months after the first visit).

**Table 1:** Study eligibility criteria

Inclusion criteria	Exclusion criteria
Adult subjects ≥ 18 years old	Pregnant women, or women in fertile age not having efficacious contraception during the whole period of the study
Able to provide informed consent to participate in the study	History of alcohol or drug abuse within the past 6 months as self-reported
Able to self-report pain	Unstable medical conditions (e.g. uncontrolled diabetes, uncompensated cardiac issues, heart failure or chronic obstructive pulmonary disease)
To have a recent diagnosis (less than 3 months) of cancer affecting lung, breast, or colon (with metastases or not)	History of neurosurgery, traumatic brain injury with loss of consciousness, and/or cortical lesions
To have not started any systemic treatment	History of nonmalignant chronic pain

**Fig. 1:**
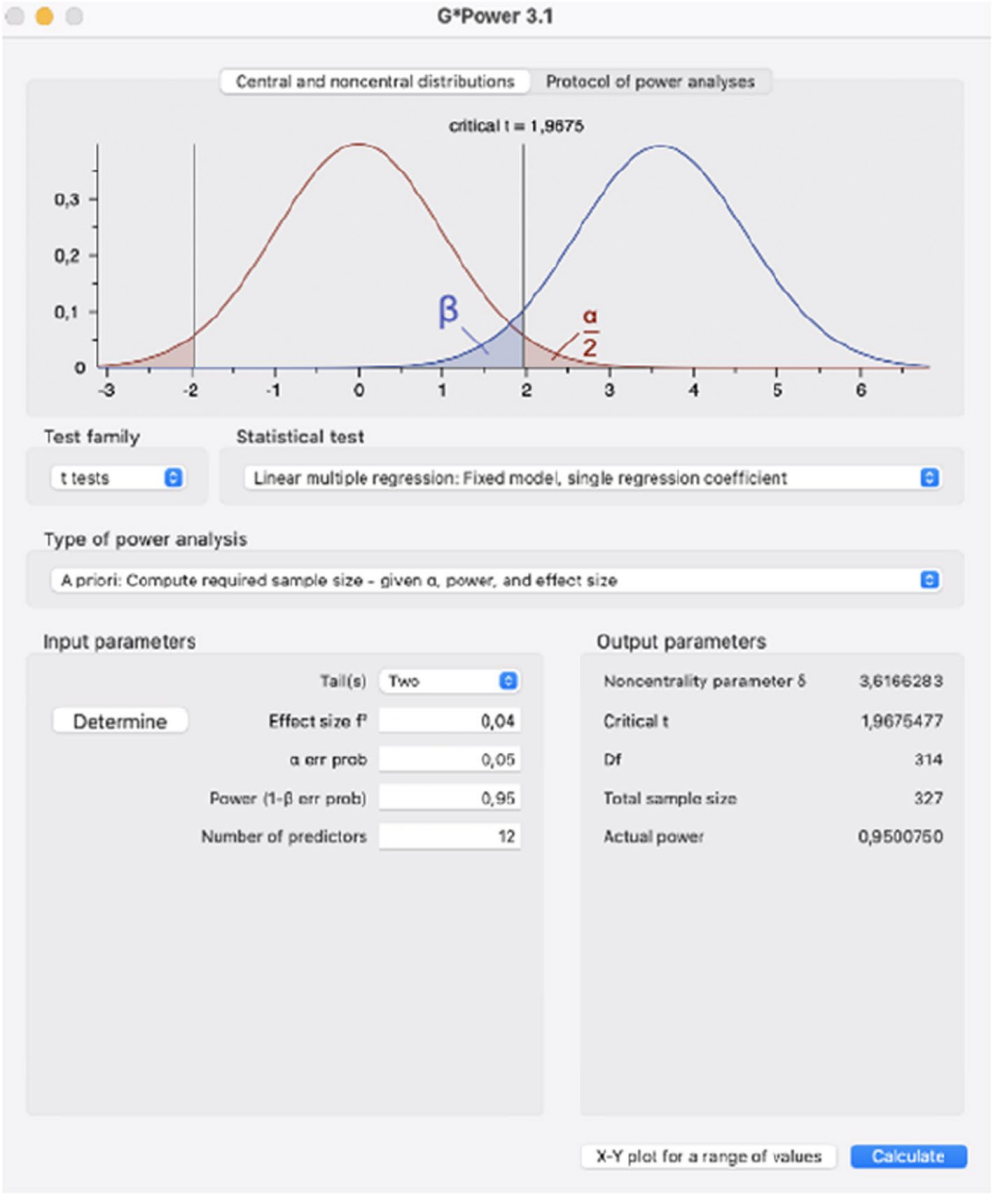
Calculation of sample size

Using GPower (v.3.1.9.7), setting alpha at 0.05 and power (1-ß) at 0.95, we estimated a final sample of 327 participants to get a two-tail small effect size of f^2^ = 0.04 in a linear multiple regression model with 12 predictors (age, sex, lifestyle, cancer type, risk-factors, comorbidities, emotional functioning, thermal pain thresholds, conditioned pain modulation, temporal summation of pain, and evoked potentials (Fig. 1). We will recruit 450 participants to compensate for the eventual dropouts in the longitudinal study (approximately 30%).

### Patient recruitment method

The five research units involved in this clinical study will recruit 450 patients (225 male, 225 female). All patients will be assessed through the procedures established for QST and EEG evaluations. Moreover, the assessments will include questionnaires and self-report measures to collect data regarding demographic and clinical variables.

Patients will be preselected daily by a clinician from the multidisciplinary team. Then, they will be contacted by a research member who will present the study protocol and invite them to complete the assessment procedures. To participate, the eligible individuals agreed to the study conditions and provided written informed consent at the three evaluation time points. The recruitment started in 2023 and will be finished at the completion of the full data collection of 450 patients.

### Data collection

Each participant will be assessed at three different time points: pretreatment, 6 months, and 12 months after the first visit. After signing the informed consent, participants will complete an exhaustive assessment protocol that includes an initial interview, the QST (pain thresholds, CPM, TSP), EEG and CHEPs and self-report measures about their health condition. During the interview, we will collect relevant sociodemographic data to characterize the sample and data from the clinical history. Each assessment session will last an estimated 2 h.

Multiple efforts have been made by various experts to prioritize and standardize outcomes in pain research. The Initiative on Methods, Measurements and Pain Assessments in Clinical Trials (IMMPACT) established six core outcome domains: 1) pain; 2) physical functioning; 3) emotional functioning; 4) participant ratings of improvement and satisfaction with treatment; 5) symptoms and adverse events; and 6) participant disposition. We will select tools to cover all those domains and perform comprehensive sociodemographic (age, sex, lifestyle, between others) and clinical assessments (comorbidities, type of tumor, extension, time since diagnosis, evolution, previous chronic diseases, risk factors for cancer, and antecedents of cancer).

#### Questionnaires and self-report measures

Based on the IMMPACT-II consensus meeting, participants will complete the following questionnaires, which were previously translated and validated in their language, using the PAINLESS platform:

- *Numeric Rating Scale (NRS) to assess pain intensity and distress*: An 11-point NRS will be used to measure the pain intensity and distress caused by pain (in the last week). The NRS score ranges from 0 (“No pain”) to 10 (“Pain as bad as you can imagine”) [7].
- *Brief Pain Inventory (BPI)*: The BPI is a tool for assessing clinical pain, allowing patients to rate the intensity and severity of their pain, as well as the degree to which their pain interferes with several aspects of life. Interference is divided into activity and affective subdimensions. A 7-day or 24-h recall period may be used. The shorter version consists of 12 items that assess two factors: the severity of pain and its impact on daily life. The severity factor queries current symptoms, symptoms on average, and the range of pain intensity that they experience. The impact factor asks respondents how pain interferes with their general activity, mood, mobility, work, relationships, sleep, and enjoyment of life. The scale is a self-report measure that requires approximately 5 min for administration [4].
- *Pain Catastrophizing Scale (PCS)*: The PCS is a 13-item measurement tool developed to help quantify patients’ pain experience, asking about how they feel and what they think about when they are in pain. Pain catastrophizing is characterized by the tendency to magnify the threat value of a pain stimulus and to feel helpless in the presence of pain, as well as by a relative inability to prevent or inhibit pain-related thoughts in anticipation of, during, or following a painful event [27]. Patients are asked to indicate the degree to which they have several thoughts and feelings when they are experiencing pain using the 0 (not at all) to 4 (all the time) scale. A total score is obtained (ranging from 0–52), along with three subscale scores assessing rumination, magnification, and helplessness. A total PCS of 30 represents a clinically relevant level of catastrophizing [35].
- *Medical Outcomes Study (MOS) 36-item Short-Form Health Survey (SF-36)*: The MOS SF-36 is a measure of health-related quality of life, defined as the extent to which physical health impacts patients’ functional ability and perceived well-being in mental, social and physical aspects of life. It has 8 individual subscales divided across physical and psychological health-related quality-of-life domains: physical function (PF; 10 items), role physical (RP; 4 items), bodily pain (BP; 2 items), general health (GH; 5 items), vitality (V; 4 items), social function (SF; 2 items), role emotional (RE; 3 items) and mental health (MH; 5 items). Scores on these subscales can be combined to form 2 higher-order summary scores, the physical component summary (PCS) and mental component summary (MCS). The PCS is calculated by positively weighting the 4 subscales in the physical domain (PF, RP, BP, and GH) and the remaining psychological domain subscales negatively. In contrast, the MCS is calculated by positively weighting the 4 mental domain subscales (MH, V, SF, and RE) and negatively weighting the 4 physical domain subscales. Likert scales and yes/no options are used to assess function and well-being on this 36-item questionnaire. Our data were collected over a 4-week time frame [42].
- *EQ-5D-3 L*: The EQ-5D-3 L is a 3-level version of the EQ-5D that was introduced by the EuroQol Group (EQ). It comprises the EQ-5D descriptive system and the EQ visual analog scale (EQ VAS). The EQ-5D-3 L descriptive system assesses mobility, self-care, usual activities, pain/discomfort, and anxiety/depression. Each dimension has 3 levels (no problems, some problems, and extreme problems). The patients are asked to indicate their health status in each of the five dimensions. This decision results in a 1-digit number that expresses the level selected for that dimension. The digits for the five dimensions can be combined into a 5-digit number that describes the patient’s health status. The EQ VAS records patients’ self-rated health on a vertical visual analog scale where the endpoints are labeled ‘Best imaginable health state’ and ‘Worst imaginable health state’. The VAS can be used as a quantitative measure of health outcomes that reflects patients’ own judgment [14].
- *Modified Fatigue Impact Scale (MFIS)*: The MFIS is a modified form of the Fatigue Impact Scale [11] based on items derived from interviews with multiple sclerosis patients concerning how fatigue impacts their lives. This instrument provides an assessment of the effects of fatigue on physical, cognitive, and psychosocial functioning. The MFIS structured, self-report questionnaire consists of 21 items. The total score for the MFIS is the sum of the scores for the 21 items. Individual subscale scores for physical, cognitive, and psychosocial functioning can also be generated by calculating the sum of specific sets of items [29].
- *Patient Health Questionnaire (PHQ-9)*: The PHQ is a self-administered instrument for making criteria-based diagnoses of depressive and other mental disorders commonly encountered in primary care. The PHQ-9 is the depression module from the full PHQ, which scores each of the 9 DSM-IV (Diagnostic and Statistical Manual of Mental Disorders – version IV) criteria. Major depression can be diagnosed if 5 or more of the 9 depressive symptom criteria have been met for at least “more than half the day” in the past 2 weeks, and 1 of the symptoms is a depressed mood or anhedonia. Minor depression can be diagnosed if 2, 3, or 4 depressive symptoms have been present for at least “more than half the day” in the past 2 weeks, and 1 of the symptoms is a depressed mood or anhedonia. Each of the 9 items can be scored from 0 (not at all) to 3 (nearly every day), and the PHQ-9 total score ranges from 0 to 27 [18].
- *Generalized Anxiety Disorder Assessment (GAD-7)*: The GAD-7 consists of 7 items asking about recent symptoms (i.e., in the past 2 weeks) of the DSM-IV criteria for generalized anxiety disorder. Each item is scored as 0 (not at all), 1 (several days), 2 (more than half the day), or 3 (nearly every day). The GAD-7 total score ranges from 0 to 21. A score of 10 or greater represents a reasonable cutoff point for identifying patients with GAD. Cutoff points of 5, 10, and 15 might be interpreted as representing mild, moderate, and severe levels of anxiety, respectively [32].
- *Sleep Scale (SS) from the Medical Outcomes Study (MOS-SS)*: The MOS-SS is a 12-item self-report measure used to assess six factors of sleep: sleep disturbance (3 items), snoring (1 item), waking short of breath or with headache (1 item), quantity of sleep (1 item), optimal sleep (1 item), sleep adequacy (2 items), and somnolence (3 items). Two additional questions were asked to assess how long the patient usually fell asleep and how many hours s/he slept each night. Each item asks about recent past (i.e., the average of the past 4 weeks) and may be answered on a 6-point Likert scale (1 meaning “All of the time” and 6 meaning “None of the time”). Optimal sleep is a dichotomous variable coded as 1 if the patient reported 7 or 8 h of sleep per night on the quantity of sleep 1-item subscale or 0 otherwise. The sleep quality score relates to the average number of hours slept (0–24). The other five subscales are linearly transformed to a 0–100 scale, with higher scores representing more of the sleep concept being measured. In addition, sleep index measures may also be constructed to provide composite scores [12].
- *Eastern Cooperative Oncology Group (ECOG) Performance Status Scale*: The ECOG describes a patient’s level of functioning in terms of their ability to care for themselves, daily activity, and physical ability (walking, working, etc.). Its scale and criteria are used by doctors and researchers to assess how a patient's disease progresses, assess how the disease affects the daily living abilities of the patient, and determine appropriate treatment and prognosis. The ECOG score ranges from 0 (fully active) to 5 (dead) [23].

#### Quantitative sensory testing (QST) biomarkers

##### Thermal pain thresholds

The threshold of thermal pain will be measured with the *Limits* method using a thermal contact stimulator (TCS II) developed by the QST. LAB (https://www.qst-lab.eu/tcs-technical-description). The TCS II stimulator enables precise measurement of the threshold due to its ability to reach the target temperature in a few milliseconds. Indeed, this device is made of several microPeltier thermoelectric elements that achieve very steep temperature ramps (i.e., up to 300 °C/s). In this way, the time required to estimate the pain threshold with this method is less than 2 min per skin area. We can thus easily activate small, thermal nociceptive nerve fibers conveyed by the spinothalamic tract. These properties yield a cost-effective test with reduced test–retest variability, fewer safety precautions, and a lower burn risk than laser stimulators.

The stimulation site will be the dominant volar forearm, which will also be used for temporal summation of pain (TSP), conditioned pain modulation (CPM), heat pain threshold (HPT), cold detection threshold (CDT), cold pain threshold (CPT), contact heat evoked potentials (CHEPSs) during EEG recording, and offset analgesia (OA).

The nondominant volar forearm will be used for familiarization, and the pain-5 level will be obtained to avoid sensitization of the dominant forearm. For resting-state EEG recording during cold pain, we used the palm of the dominant hand.

If the volar forearm and palm of the dominant arm of the hand are the primary painful sites for a given patient, the procedure should be applied to the nondominant arm counterparts. If this is also considered a primary site of pain, the dorsum of the forearm or hand will be used instead.

The perceived pain intensity in response to ongoing thermal stimulation will be assessed using a numerical rating scale (NRS) ranging from 0 to 10. Anchor point ‘0’ is defined as ‘no pain’, and anchor point ‘10’ is the ‘worst pain imaginable’ by the participant. The procedure will start with familiarization, a short training session to familiarize participants with painful stimuli. The stimulation will be applied to the *nondominant* volar forearm (heat pain: 1 stimulus of 45 °C for 5 s). Ramp-up and ramp-down: 170 °C/s; cold pain: 1 stimulus of 0 °C for 5 s. Ramp-up and ramp-down: 170 °C/s). The baseline temperature will be 32 °C.

Then, the Pain-5 score, defined as the temperature resulting in a pain intensity rating of 5 on the NRS, was recorded. Two determination procedures will be repeated twice (each): i) Pain-5 for TSP and ii) Pain-5 for CPM and offset analgesia (OA). Both pain-5 measurements were calculated as the mean of the two repetitions for each determination procedure. For each procedure, we will start with the first stimulation at 45 °C. If the pain intensity rating is higher than 5/10, we will reduce the temperature by -1 °C (i.e., 44 °C) for subsequent stimulation. Conversely, if the pain intensity rating is lower than 5/10, the temperature will increase by + 1 °C (i.e., 46 °C) for the next stimulation. We will repeat this process until a pain intensity rating of 5/10 is reached. We will follow the next sequence:

- *First repetition of Pain-5 for TSP*: The stimulation duration will be 0.5 s, and the interstimulus interval will be 5 s;
- *First repetition of Pain-5 for CPM and OA*: The stimulation duration will be 10 s, and the interstimulus interval will be 5 s;
- *Second repetition of Pain-5 for TSP*: The stimulation duration will be 0.5 s, and the interstimulus interval will be 5 s;
- *Second repetition of Pain-5 for CPM and OA*: The stimulation duration will be 10 s, and the interstimulus interval will be 5 s.

The interval between each Pain-5 repetition (a, b, c, and d) will be 90 s. The stimulation site on the nondominant volar forearm should be moved slightly between each stimulation to avoid habituation of the thermal receptors. The baseline temperature will be 32°C. The Pain-5 for TSP will be calculated as the mean of the temperature yielding a pain intensity rating equal to 5/10 for repetitions a and c, while the Pain-5 for CPM and OA will be calculated as the mean of the temperature yielding a pain intensity rating equal to 5/10 for repetitions b and d.

##### Temporal summation of pain (TSP)

The TSP will also be evoked with the TCS II stimulator, and stimulation will be applied to the dominant volar forearm. Initially, a single stimulus at the determined “Pain-5 for TSP” temperature will be applied, with a pulse duration of 0.5 s and a ramp up and down of 170 °C/s. The patient will be asked to rate the pain intensity for this stimulus (time to rate 10 s). Then, 10 stimuli at the determined “Pain-5 for TSP” temperature will be applied with a pulse duration of 0.5 s, a ramp up and down of 170 °C/s, and an interstimulus interval of 0.5 s (i.e., stimulus onset to onset interval of 1 s and stimulation frequency of 1 Hz). The participant will be asked to rate the pain intensity for the last of the stimuli in the sequence. The baseline temperature is set to 32 °C.

The TSP index will be calculated as the normalized difference in pain intensity ratings between the initial single stimulus and the last pulse of the 10-stimulus sequence. After the TSP, patients will be given a break of 60 s.

##### Conditioned pain modulation (CPM)

CPM requires the use of two different stimuli: i) the test stimulus (TS) is a “Pain-5 for CPM and OA” temperature applied with a 10 s duration and a ramp-up and ramp-down of 170 °C/s in the dominant volar forearm; and ii) the conditioning stimulus (CS) is applied by having the participant rest the palm of their nondominant hand on a cold plate at 10 °C for 40 s.

First, the TS will be applied, and participants will be requested to rate the evoked pain intensity on the NRS. Then, participants will be asked to place the palm of their nondominant hand on the cold plate. When the hand has been on the cold plate for 20 s, the participant will be asked to rate the pain intensity of this stimulation using the NRS. When the hand has been on the cold plate for 30 s, the TS will be reapplied, and the participant will be asked to rate the pain intensity evoked by this TS stimulus using the NRS. As soon as the evoked pain intensity for this second TS application (i.e., “conditioned TS”) has been produced, the palm of the nondominant hand is removed from the cold plate. The TS stimulation site should be moved slightly for each TS application to avoid habituation of the thermal receptors. The baseline temperature will be 32 °C.

The CPM index will be calculated as the normalized difference between the evoked pain intensity ratings for the “conditioned TS” and the first application of the TS (i.e., “unconditioned TS”). After the CPM, patients will be given a break of 120 s.

##### The heat pain threshold (HPT), cold detection threshold (CDT) and cold pain threshold (CPT)

These thresholds will be estimated using the method of limits as described by Rolke et al. [30]. For each threshold (HPT, CDP, and CPT), we will perform 3 trials, with 5 s intervals between them. For the HPT and CPT estimation, the participant will be asked to press the response button as soon as a hot or cold sensation, respectively, becomes painful. For the CDT estimation, the participant will be asked to press the response button as soon as cold is perceived.

The stimulation site will be the dominant volar forearm and will be moved slightly between each stimulation to avoid habituation of the thermal receptors. The baseline temperature will be 32 °C. Temperature changes (i.e., increases for HPT and decreases for CDT and CPT) will occur at a rate of 1 °C/s. Between the HPT and CPT trials (i.e., as soon as the response button is pressed), the temperature will return to the baseline level at a rate of 8 °C/s. For CDT, it will return at a 170 °C/s rate.

The arithmetic means of the temperature at response button press across the 3 trials for each threshold will be used as the corresponding threshold estimation. The sequence of application will be as follows: HPT (120 s rest); CDT; and CPT.

##### Offset analgesia (OA)

OA is defined as a disproportional decrease in perceived pain intensity after a slight decrease in a painful heat stimulus [39]. First, a familiarization will be conducted with the stimulation intensity set at the determined “Pain-5 for CPM and OA” temperature minus 2 °C for 10 s, delivered to the dominant volar forearm. Participants will be asked to use an electronic visual analog scale (eVAS) to rate the perceived pain intensity evoked by this stimulation. OA will be induced by applying i) the determined Pain-5 temperature for CPM and OA for 5 s; ii) this temperature plus 1 °C for another 5 s; and iii) the original Pain-5 temperature for 20 s. A constant heat stimulus with the determined Pain-5 for CPM and OA temperature for 30 s will be used as a control stimulus. It will be applied either after or before the OA stimulus according to a counterbalancing of the sample scheme. The patients will rate the perceived pain intensity continuously on the eVAS during both stimulation paradigms (OA and control). The baseline temperature will be 32 °C. The OA index was calculated as the normalized difference in pain intensity ratings during the last 20 s of the OA and control trials.

#### Brain electrical activity (EEG and CHEPs)

EEG will allow the recording of brain activity. A portable, adaptable, and simple recording system developed and adapted for clinical use by MENTALAB will be employed. EEG signals will be recorded by 32 wet electrodes mounted in an elastic cap and arranged according to the international 10–20 system. Moreover, an electrocardiogram (ECG) recording will be performed using one electrode. The EEG signal will be amplified and digitized using a sampling rate of 250 Hz, and the reference will be in CPz. Impedances will be kept under 10 kΩ for all electrodes. The EEG session will comprise recording of brain activity under three different conditions:

a *Resting-state EEG:* We will record 5 min of spontaneous EEG with participants having their eyes closed. During this period, patients will be instructed to sit comfortably and relax, letting their mind wander.
b *Resting-state EEG during cold pain*: We will record 5 min of spontaneous EEG with participants with their eyes closed to rate how intense the painful stimulus was during the 5 min on average. The intensity of the cold painful stimulus must be between 3–5/10 on the NRS during the whole recording. To determine the required temperature, we will set the cold plate at a temperature of 20 °C and ask the participant to place the palm of their dominant hand on the cold plate for 30 s. Then, we asked the participants to rate their pain intensity on the NRS. If the pain intensity rating is higher than 5/10, we increase the cold plate temperature by 2 °C. Conversely, if the pain intensity rating is lower than 3/10, the cold plate temperature will decrease by 2 °C. We will repeat this process until the pain intensity is between 3–5/10. After the required temperature is determined, we will start the EEG recording while the patients maintain their hand on the cold plate. If the cold stimulus becomes too painful during the 5 min of the recording, the participant will be allowed to take his/her hand off the cold plate for a few seconds. EEG recording should be stopped during this period, and pain intensity ratings should be assessed. After this procedure, patients will pause for 120 s.
c *Contact heat evoked potentials (CHEPs)*: will be recorded to obtain a neural correlate of nociceptive processing with high temporal resolution. Heat stimulation will be applied to the dominant volar forearm using the TCS-II device. The stimulation location will be moved slightly between each stimulus application to avoid habituation of the thermal receptors. We will apply 20 60 °C stimuli with a duration of 0.3 s and ramp-up and ramp-down speeds of 170 °C/s, with an interstimulus interval of 10 s, to minimize saliency and startle responses. The baseline temperature will be 32 °C. If the stimulus is very painful, the TCS-II temperature should be reduced by -1 °C. This process might be repeated until 55 °C is reached. At the end of the test, the participant rated how painful the stimuli sequence was on average on an NRS. The amplitudes of the N1-P1 complex and the N2 and P2 components during heat painful stimulation, as well as time–frequency analysis of the EEG signals, will be the focus of data processing. Quantitative EEG (qEEG) analytical techniques will enable the evaluation of changes in spectral power and intersite phase connectivity [6].

### Planned statistical analyses

Statistical analyses and AI algorithms will be developed and tested by SIMAVI. We will use conventional and data-driven statistical analyses to test the predictive power of the biomarkers of central pain modulation to stratify patients.

With respect to conventional statistical methods, we will use regression analyses to test the power of biomarkers and moderator variables to predict the occurrence of pain. The biomarkers QST and EEG indices will be investigated as predictors of posttreatment chronic pain, while sociodemographic (age and sex), affective (anxiety and depression) and clinical variables (comorbidities, time since diagnosis, extension of the tumor, type of treatment, risk factors for cancer, etc.) will be investigated as moderators of the regression models.

Within the data-driven analysis, we will use a novel methodological approach to select the best biomarker classifiers and profile patients using supervised machine learning algorithms (see http://scikit-learn.org/stable/modules/svm.html).

## Discussion

The nature of this study is mainly exploratory. Given the data in the literature, the authors expect to observe chronic pain at 6 and 12 months after cancer treatment in patients:

- A pattern of increased excitability to nociceptive stimuli, with increased amplitude of TSP.
- Limited central analgesic regulation is associated with a lower CPM.
- Increased/modified brain electrical responsiveness (increased amplitudes of CHEP components) to nociceptive stimulation.
- Modifications in oscillatory electrical activity (in frequencies such as theta, alpha, and gamma frequencies) in areas such as the operculo-insular cortex and primary somatosensory cortex.
- Increased subjective pain levels.

We also hypothesize that patients will be categorized into two large groups: 1) patients with defective central endogenous pain mechanisms and 2) patients with normal central endogenous pain mechanisms. Additionally, we explored the role of other variables, such as age, sex, cancer diagnosis, and type of treatment.

This study is an original longitudinal cohort of oncologic patients, with a set of comprehensive measures, including demographic, clinical, behavioral, psychosocial, sensorial, and neurophysiological variables, that are well recognized and internationally validated. This research will provide valuable information in one of the most promising fields of medicine: cancer pain.

With the collected data from this study, it will be possible to achieve a better understanding of chronic oncological pain and the factors that contribute to its occurrence. Using AI algorithms to characterize and profile patients based on central pain modulation/processing biomarkers and moderator variables (sociodemographic and clinical variables), we expect to create a tool (based on AI algorithms) to predict the occurrence of pain. This could be very useful for making therapeutic decisions. In addition, understanding the mechanisms underlying oncological pain can contribute to the stratification of patients and their personalized treatment, improving the management of chronic cancer pain.

## Data Availability

Not applicable.

## Abbreviations

SF-36: 36-item Short-Form Health Survey
AI: Artificial intelligence
BP: Bodily pain
BPI: Brief Pain Inventory
CDT: Cold detection threshold
CHEPs: Contact heat-evoked potentials
CPM: Conditioned pain modulation
CPT: Cold pain threshold
CS: Conditioning stimulus
DSM-IV: Diagnostic and Statistical Manual of Mental Disorders – version IV
ECOG: Eastern Cooperative Oncology Group Performance Status Scale
EEG: Electroencephalography
EQ: EuroQol Group
EQ VAS: EQ visual analog scale
eVAS: Electronic visual analog scale
GAD-7: Generalized Anxiety Disorder Assessment
GH: General health
HPT: Heat pain threshold
IMMPACT: The Initiative on Methods, Measurements and Pain Assessments in Clinical Trials
MFIS: Modified Fatigue Impact Scale
MH: Mental health
MOS: Medical Outcomes Study
MOS-SS: Sleep Scale (SS) from the Medical Outcomes Study
NRS: Numeric Rating Scale
OA: Offset analgesia
PCS: Pain Catastrophizing Scale
PF: Physical function
PHQ-9: Patient Health Questionnaire
qEEG: Quantitative EEG
QST: Quantitative sensory testing
RE: Role emotional
RP: Role physical
SF: Social function
TCS II: Thermal contact stimulator
TS: Test stimulus
TSP: Temporal summation of pain
V: Vitality
